# Relations of Chemistry to Dentistry

**Published:** 1897-11

**Authors:** J. S. Cassidy

**Affiliations:** Covington, Ky.


### COMMUNICATIONS.

### Relations of Chemistry to Dentistry.

BY J. S. CASSIDY, M.D., D.D.S., COVINGTON, KY.

Abstract of Paper read before the American Dental Association, Old Point Comfort, Va., Aug. 1897.

Dentistry is and should be cosmopolitan. It appropriates with due permission facts and methods from the humblest as well as the most exalted occupations, and in return gives back to them a large percent of the benefits received. Metallurgy, molding, sculpture, surgery, medicine, physics and chemistry have been appealed to not in vain; and as a consequence the accomplished dentist could point out to willing youth the best means of becoming experts in these separate vocations.

Of all these sciences that of chemistry is the most universal, since it has no limitations; all forms of matter and force must own its sway. To chemistry our profession is indebted in that it prepared $N_2O$, by which means in after years, Dr. Horace Wells, a dentist, might be permitted to introduce anesthesia, the greatest boon ever conferred on suffering humanity.

Dentists, as a rule, are not indifferent to, nor ignorant of chemical processes; notwithstanding our friend, Dr. Crouse, asserted there was not a single dentist in this country who could make an accurate quantitative analysis of any dental amalgam; whereas hundreds might be named who could do so, if it were necessary. Indeed, there are many men, not professional chemists, who are able to do excellent work along lines of chemical discovery and manipulation. The recent studies of the chemical and physical properties of amalgams are of inestimable value to every dentist who thankfully takes advantage of the information derived through the labors of Dr. Black.

Moreover not to mention the innumerable pharmaceutical preparations, our zinc oxide cements may be brought in evidence; and as some of us are sure that fillings of these materials are disposed to disintegrate more at the cervical point than elsewhere, we also think we know the true and only cause of this unfortunate disposition, in that the part under or near the gum margin is the favorite point for alkalin fermentation, such as the production of ammonia, resulting in the inevitable abstraction by it of the electro-negative substance of the cement. Some may be inclined to consider this brief allusion to cause and effect as "only a fine spun theory," but it is not; it is as susceptible of proof as any problem in mathematics.

We can not, as dentists, escape, even if we desire, the claims of the science of chemistry upon our earnest attention and study, for on every hand we are compelled to admit the insidious influence of its affinities to be the exciting and efficient cause of the principal disease with which we have to contend. Happily, the study of chemistry, while acquainting us with these potent influences, also points out to us within the sphere of the same science the means at hand to combat them, most beneficially provided, if we will but apply them.

No member of this—as the late Dr. Atkinson termed it—"Excelsior Association of Dentists" will doubt the culminating discovery of Dr. Miller, that at least lactic acid is developed in the mouth by the presence of bacteria and the materials necessary to their support, and also to the play of chemical affinities at the point of carious destruction; and, further, that the destroying agent act molecularly with as definite results as pertain to any other natural phenomenon. Some years ago, when the so-called "germ theory" of disease was introduced, there was not a few who received the innovation with satisfaction; because they would not or could not understand, and therefore approve, the chemical theories of Watt. It is now, however, accepted beyond question that there is no conflict between the two theories—that bacteria are a necessary factor in fermentation, and that their only trysting-places are in the midst of extraneous material susceptible to that process, the elements of which must obey the laws of their affinities; and if, for instance, among other compounds, destructive and otherwise, lactic acid, as proven by Miller, is developed in contact with a tooth, destruction of that part will proceed molecule by molecule; so that, while it is probable that teeth, like other organs, yield to an increased non-resisting influence of a "periodic law," dental caries is *per se* a disease of purely chemical propagation. From this latter view-point the relations between chemistry and dentistry are not of the most amicable character, yet the science whose unseen minions are directly responsible for the exigency of our principal enemy will inevitably furnish weapons of defense other than we as yet possess.

No wonder that dentists are perhaps unconsciously more interested in chemistry than are the members of the mother profession; at least, it would so appear by personal contact with them, and by impartial reading of medical and dental journals. One of the former recently, to cite a single example, expressed its surprise that any two acids could neutralize each other; and then went on to say, that by mixing one part of acetic acid, and one and one-half of carbolic acid, a neutral compound will result. Any tyro in organic chemistry knows that phenol is not an acid, but on the contrary is an alcohol and that an alcohol and an acid combined will produce an ester, and water:

$$\underset{\text{Phenol}}{C_6 H_5 HO} + \underset{\text{Acetic acid}}{C_2 H_4 O_2} = \underset{\text{Phenyl acetate}}{C_6 H_5 C_2 H_3 O_2} + H_2 O$$

It goes without saying that a foreknowledge of certain rules in chemistry is a great aid in adapting means to ends, when anything new in that line appears. For instance, in cataphoresis, we were told at first that the positive pole must be used in contact with whatever drug was employed; whereas it is well known in electrolysis that the radicals or ions of conducting-compound liquids separate in a perfectly definite manner, the positive radical being attracted to the cathode and the negative to the anode; so that we will likely obtain better results by selecting anaphoresis when we wish greater penetration of a strongly electro-negative radical. A duly certified list of comparative positive and negative classes of radicals has been known for many years, of which we are welcome to take advantage; and in return for all these favors, would it not be an act of courtesy on our part to approve, when occasion permits, certain changes in chemical nomenclature, adopted through official international committees appointed for the purpose, by the most influential scientific bodies? Our journals, it is almost unnecessary to say, editorially are fully up to, indeed, in advance of the times, but we see too often in reports of discussion in our local societies, and in some of our latest textbooks, names of things and terms that shock the sensibilities of euphony and truth. Take for example, *potassium iodid*. Is not that name more pleasant than iodid of potash, especially when we realize that the compound contains no potash whatever?

We have been surfeited for a long time with "cocain hydrochlorate," and lately with "eucain hydrochlorate," although a generation ago it was decided that acids like hydrochloric (HCl), hydrobromic (HBr), etc., whose electro negative radicals are elementary, confer names on salts which terminate in the *ide* (the final "e" has been eliminated); therefore the name of every salt of which acids should end in "id," as "cocain hydrochlorid." Only those acids which have compound negative radicals, give names to salts ending in "ate" or "ite," as the case may be. Sulfuric acid ($H_2SO_4$) forms sulfates; sulfurous acid ($H_2SO_3$) sulfites; chloric acid ($HClo_3$) chlorates; chlorous acid ($HClo_2$) chlorites, etc.

Life is too short to nominate for consideration at this time more than one other frequently misapplied word, which is "density." We use it, do we not? as a synonymn for hardness in the structural substance of teeth, instead of in the true meaning, i. e., specific gravity. Ice is harder and more compact to a cutting instrument than is liquid water, but it is less dense. The diamond, among the hardest of bodies known, is only three and one-half times as heavy as water; while pure gold is comparatively soft, although more than nineteen times as heavy as the standard, water. It is not possible to believe that the specific gravity of a tooth has anything to do with predisposition to disease, or even that compactness of calcium constituents is a condition that presents a physiological barrier to decay. While these matters may perhaps appear of small practical importance, yet surely, as we claim to be devoted disciples of pure science, we have no right to trifle with the accepted nomenclature.

